# Impact of the COVID-19 pandemic on perinatal care and outcomes: A retrospective study in a tertiary hospital in Northern Ghana

**DOI:** 10.1371/journal.pone.0301081

**Published:** 2024-05-31

**Authors:** Alhassan Abdul-Mumin, Kingsley Appiah Bimpong, Cesia Cotache-Condor, Jonathan Oppong, Ana Maria Simono Charadan, Adam Munkaila, Joao Vitor Perez de Souza, Emily R. Smith

**Affiliations:** 1 Department of Pediatrics and Child Health, Tamale Teaching Hospital, Tamale, Ghana; 2 Department of Pediatrics and Child Health, School of Medicine, University for Development Studies, Tamale, Ghana; 3 Department of Surgery, Duke School of Medicine, Durham, North Carolina, United States of America; 4 Duke Global Health Institute, Duke University, Durham, North Carolina, United States of America; 5 Center for Global Surgery and Health Equity, Duke University, Durham, North Carolina, United States of America; 6 Department of Obstetrics and Gynecology, Tamale Teaching Hospital, Tamale, Ghana; 7 Department of Obstetrics and Gynecology, School of Medicine, University for Development Studies, Tamale, Ghana; 8 Division of Translational Health Sciences, Department of Emergency Medicine, Duke School of Medicine, Duke University, Durham, North Carolina, United States of America; University of Ghana Medical School, GHANA

## Abstract

**Background:**

Perinatal mortality remains a global challenge. This challenge may be worsened by the negative effects of the COVID-19 pandemic on maternal and child health.

**Objectives:**

Examine the impact of the COVID-19 pandemic on perinatal care and outcomes in the Tamale Teaching Hospital in northern Ghana.

**Methods:**

A hospital-based retrospective study was conducted in the Tamale Teaching Hospital. We compared antenatal care attendance, total deliveries, cesarean sections, and perinatal mortality before the COVID-19 pandemic (March 1, 2019 to February 28, 2020) and during the COVID-19 pandemic (March 1, 2020 to February 28, 2021). Interrupted time series analyses was performed to evaluate the impact of the COVID-19 pandemic on perinatal care and outcomes at TTH.

**Results:**

A total number of 35,350 antenatal visits and 16,786 deliveries were registered at TTH from March 2019 to February 2021. Antenatal care, early neonatal death, and emergency cesarean section showed a rapid decline after the onset of the pandemic, with a progressive recovery over the following months. The total number of deliveries and fresh stillbirths showed a step change with a marked decrease during the pandemic, while the macerated stillbirths showed a pulse change, a temporary marked decrease with a quick recovery over time.

**Conclusion:**

The COVID-19 pandemic had a negative impact on perinatal care and outcomes in our facility. Pregnancy monitoring through antenatal care should be encouraged and continued even as countries tackle the pandemic.

## Introduction

Perinatal mortality remains a major challenge globally. More than 90% of these deaths are reported in low-and middle-income countries (LMICs) [1,2]. An estimated 41% of under-5 deaths occur in the neonatal period [3], and about three quarters of these deaths occur in the first week of life [4]. The majority of these deaths are attributed to preventable conditions, such as infections, prematurity, congenital anomalies related to malnutrition, and low birth weight. These conditions are frequently exacerbated by poverty, lack of adequate prenatal care, and poor health infrastructure [5,6].

Globally, there has been a decline in neonatal mortality from an estimated 37 per 1000 live births in 1990 to 18 per 1000 live births in 2018, with a 52% decline over this period. sub-Saharan Africa (SSA) has lagged behind this progress with only an estimated 39% decline noted over this period [7].

Similar trends have been observed in stillbirth rates with an estimated 2.6 million stillbirths recorded globally, in 2015. Global estimates show a 25.5% reduction in stillbirths between the periods of 2000–2015, with SSA having only an estimated 19.4% decline over this time period [8]. A study in a Municipal hospital in Ghana reported a decrease in stillbirths of 4.2% to 2.1% over the 10 year period of the study [9].

It is feared that the gains made in perinatal care may be derailed by the negative indirect effects of the COVID-19 pandemic [10,11]. Similar indirect effects on maternal, neonatal and child health were documented in the Ebola hemorrhagic disease outbreak in west Africa [12,13]. This might be explained by late presentations and refusal to access maternity care because of fear of contracting the virus [14,15]. Early modelling studies projected dire indirect effects of the COVID-19 pandemic on maternal and child health, with increase in maternal and child deaths in LMICs [16]. A study conducted in Nepal, reported an increase in stillbirth rate from 14 to 21 per 1000 births, and neonatal deaths from 13 to 40 per 1000 live births during the lockdown [17]. Likewise, a threefold increase in stillbirths were reported in a study in Italy [18]. Furthermore, there has been extensive negative impact on the provision of maternity services, with reported decrease in antenatal appointments [18,19].

Although Ghana is one of the worst affected countries in SSA with a total case count of 131,412 infections and 1,239 deaths as of December 12, 2021 [20], no study has looked yet at the effect of the pandemic on perinatal care and outcomes in northern Ghana. This retrospective study aims to compare perinatal mortality and care in a tertiary hospital in Ghana before and during the COVID-19 pandemic and builds on our current work to improve children’s care in northern Ghana [6]. As the country is faced with the prospects of a fourth wave of the pandemic, our findings could inform decisions to improve interventions and policies.

## Materials and methods

### Study setting and participants

The Tamale Teaching Hospital (TTH) is a tertiary level facility located in the northern part of Ghana. It conducts about 8,000 deliveries per annum. As a teaching hospital, TTH serves as a center of training for clinical students at the University for Development Studies School of Medicine, Nursing and Midwifery, and Allied Health Sciences. The institution also serves as a major referral center for patients from Northern, Savannah, Upper West, Upper East, North East, Oti and Bono East regions, and sometimes from neighboring countries: Togo, Cote d’Ivoire, Burkina Faso [21].

### Study design and data collection

This is a hospital-based retrospective study carried out at TTH to compare perinatal care and outcomes before and during the COVID-19 pandemic. Our study complies with the STROBE checklist guidelines. Data collection for this study took place During May 2021 and December 2023 by KAB and AAM. The obstetric department keeps a register of all births while the NICU keeps an electronic record of all neonatal admissions into the unit. All deliveries conducted at TTH from March 1, 2019 to February 28, 2021 were included in this study. Total monthly data for the study period was retrieved using a structured data collection tool. Identifiable data was not accessible to the researchers at any step during or after the data collection. The information included month, year, number of antenatal attendances, total deliveries, vaginal deliveries (spontaneous and assisted), stillbirths (fresh and macerated), cesarean sections (elective and emergency), and early neonatal death.

### Data analysis

We defined the pre COVID-19 era as the one-year period ranging from March 1, 2019 to February 28, 2020. The COVID-19 era was defined as the one-year period ranging from March 1, 2020 to February 28, 2021. Early neonatal death was defined as neonatal death within the first 7 days of life whereas stillbirth was defined as fetal death on or after 28 completed weeks of gestation, or with a weight >1Kg. Fresh stillbirth was defined as fetal death during delivery. Macerated stillbirth was defined as death of the fetus before onset of labor with the fetus showing degenerative changes [22]. Perinatal mortality was calculated as the sum of total stillbirth and early neonatal death. Total deliveries were calculated as the sum of live births and total stillbirths. Live births were calculated as the sum of total cesarean sections and total vaginal deliveries.

We used descriptive statistics to analyze the distribution of all perinatal care and outcomes variables across the two-year period. The Wilcoxon rank sum test was performed to compare all variables’ medians before and during the COVID-19 pandemic, with a statistical significance set at p<0.05.

We used interrupted time series (ITS) analysis with ARIMA to evaluate the impact of the COVID-19 pandemic on antenatal care, total deliveries, total cesarean sections, elective cesarean sections, emergency cesarean sections, total perinatal death, early neonatal death, fresh stillbirths and macerated stillbirths. Count data was used to assess antenatal care and total deliveries. However, we used the standardized version of the rest of the variables to reduce bias. Standardization was achieved by dividing the monthly data for each variable by the total number of deliveries in the respective month. Then, we assessed autocorrelation for each variable by examining the autocorrelation function (ACF) and the partial autocorrelation function (PACF) plots. We used the automated algorithm, auto.arima(), to identify a suitable ARIMA model for each of the variables. For all variables, the ACF/PACF plots and the auto.arima() function suggested models with no seasonality component. The Box-Ljung test and diagnostic plots were used to assess autocorrelation in the model and model fit (**S1 Fig**). Finally, counterfactual data were predicted to understand the trend each variable might have had if the pandemic never happened.

Three impact scenarios were modeled for each variable. First, a step change, defined as a sudden positive or negative sustained change that follows the onset of the pandemic. Second, a pulse change, defined as sudden and temporary positive or negative change occurring after the onset of the pandemic and is followed by a rapid recovery until reaching the baseline level. Third, a combination of a step change and a ramp change. This latter was defined as a progressive positive or negative change in the slope that follows the onset of the pandemic. For each variable, the final model was selected based on performance according to the following indicators in order of priority: statistical significance of confidence intervals, statistical significance or lower p-value, and lower Akaike information criterion (AIC) (**S1 Table**). In the sensitive analysis, all variables were modeled based on their count data instead of their standardized version. The ITS analysis was performed in RStudio 2023.12.0.

### Ethics statement

The study team obtained ethical clearance from the Ethical Review Committee of the TTH with reference number TTHERC/30/03/21/03. Participant consent was not sought due to the retrospective nature of this study.

## Results

We recorded a total of 35,350 antenatal visits and a total of 16,786 deliveries in the TTH from March 2019 to February 2021 (**Table 1**). Of the total of live births, 71.2% were vaginal deliveries and 28.8% were cesarean sections. Most of the latter were emergency cesarean sections (69.7%). The majority of perinatal deaths were fresh stillbirths (38.3%), followed by early neonatal deaths (37.7%), and macerated stillbirths (24.0%).

**Table 1:** Distribution of perinatal care and outcomes reported at TTH before and during the COVID-19 pandemic.

	Total		Before COVID-19		During COVID-19	
	*% (n)*		*% (n)*		*% (n)*	
**Antenatal care attendance**	100	(35,350)	65.7	(23,217)	34.3	(12,133)
**Total deliveries**	100.0	(16,786)	53.4	(8,963)	46.6	(7,823)
Live births	96.0	(16,117)	95.5	(8,557)	96.6	(7,560)
Stillbirths	4.0	(669)	4.5	(406)	3.4	(263)
**Live births**	100.0	(16,117)	53.1	(8,557)	46.9	(7,560)
Vaginal delivery	71.2	(11,470)	72.0	(6,163)	70.2	(5,307)
Cesarean section	28.8	(4,647)	28.0	(2,394)	29.8	(2,253)
**Vaginal deliveries**	100.0	(11,470)	53.7	(6,163)	46.3	(5,307)
Spontaneous vaginal delivery	99.7	(11,431)	99.7	(6,146)	99.6	(5,285)
Assisted vaginal delivery	0.3	(39)	0.3	(17)	0.4	(22)
**Cesarean sections**	100.0	(4,647)	51.5	(2,394)	48.5	(2,253)
Elective	30.3	(1,409)	32.5	(779)	28.0	(630)
Emergency	69.7	(3,238)	67.5	(1,615)	72.0	(1,623)
**Perinatal death**	100.0	(1,073)	57.6	(618)	42.4	(455)
Fresh stillbirth	38.3	(411)	42.7	(264)	32.3	(147)
Macerated stillbirth	24.0	(258)	23.0	(142)	25.5	(116)
Early neonatal death	37.7	(404)	34.3	(212)	42.2	(192)

When comparing these variables before and during the COVID-19 pandemic, the median distribution of antenatal visits, total perinatal deaths, and fresh stillbirths were statistically significant (**Table 2**). The median antenatal visit per month decreased by 758 units, from 1,858 to 1,100 monthly visits during the COVID-19 pandemic (p<0.001). Likewise, the median total perinatal death and fresh stillbirths per month decreased by 16 and 11 units during the COVID-19 pandemic, respectively, from 52 to 36 total perinatal deaths (p = 0.01), and from 21 to 10 monthly fresh stillbirths (p = 0.007).

**Table 2:** Distribution of perinatal care and outcomes reported at TTH before and during the COVID-19 pandemic.

Variable	Before COVID-19	During COVID-19	p-value
**Antenatal Attendance**			**<0.001**
Median (IQR)	1,858 (1,815, 2,046)	1,100 (870, 1,335)	
Mean (SD)	1,935 (197)	1,011 (486)	
Range	1,742–2,388	42–1,657	
**Total deliveries**			0.13
Median (IQR)	778 (659, 820)	608 (572, 766)	
Mean (SD)	747 (119)	652 (159)	
Range	523–902	322–864	
**Total vaginal deliveries**			0.2
Median (IQR)	524 (468, 563)	404 (382, 533)	
Mean (SD)	514 (87)	442 (136)	
Range	335–648	136–636	
**Total cesarean sections**			0.3
Median (IQR)	210 (172, 227)	184 (177, 204)	
Mean (SD)	200 (38)	188 (32)	
Range	117–241	133–258	
**Elective cesarean sections**			0.1
Median (IQR)	61 (56, 82)	54 (47, 62)	
Mean (SD)	65 (20)	52 (12)	
Range	24–91	25–68	
**Emergency cesarean sections**			0.5
Median (IQR)	144 (117, 148)	138 (114, 143)	
Mean (SD)	135 (23)	135 (27)	
Range	93–168	95–193	
**Total perinatal death**			**0.01**
Median (IQR)	52 (48, 60)	36 (33, 42)	
Mean (SD)	52 (13)	38 (8)	
Range	25–70	28–56	
**Fresh stillbirths**			**0.007**
Median (IQR)	21 (16, 25)	10 (8, 14)	
Mean (SD)	22 (9)	12 (8)	
Range	10–40	2–34	
**Macerated stillbirths**			0.7
Median (IQR)	10.5 (9.0, 13.0)	10.0 (6.5, 12.0)	
Mean (SD)	11.8 (7.8)	9.7 (3.9)	
Range	1.0–34.0	4.0–16.0	
**Early neonatal deaths**			0.6
Median (IQR)	15.5 (12.8, 21.2)	16.0 (12.8, 17.5)	
Mean (SD)	17.7 (6.6)	16.0 (5.5)	
Range	10.0–29.0	8.0–27.0	

**Fig 1:**
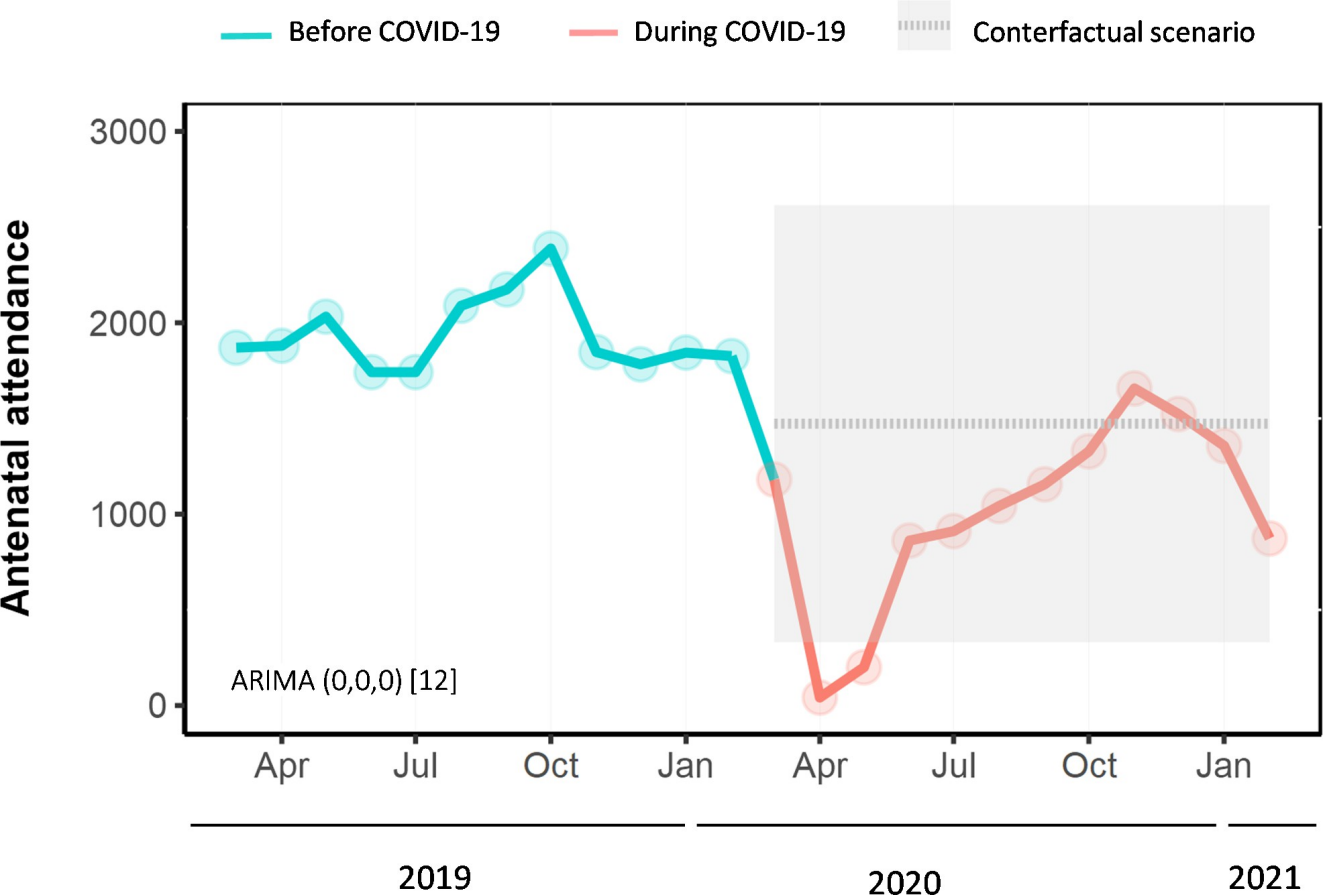
Interrupted time-series analyses of the impact of the COVID-19 pandemic on antenatal attendance (March 2019 –February 2021) at Tamale Teaching Hospital in Northern Ghana. Note: Observed antenatal attendance (solid lines) was plotted against the counterfactual monthly number of antenatal attendances predicted by the ARIMA model (grey dashed line) if the COVID-19 pandemic had not occurred. The grey shading depicts 95% prediction intervals. The numbers next to ARIMA in parentheses indicate which components have been included to generate the counterfactual (p,d,q). The square brackets indicate that the model is generated using monthly data (12 months in a year). ARIMA = autoregressive integrated moving average.

**Fig 2:**
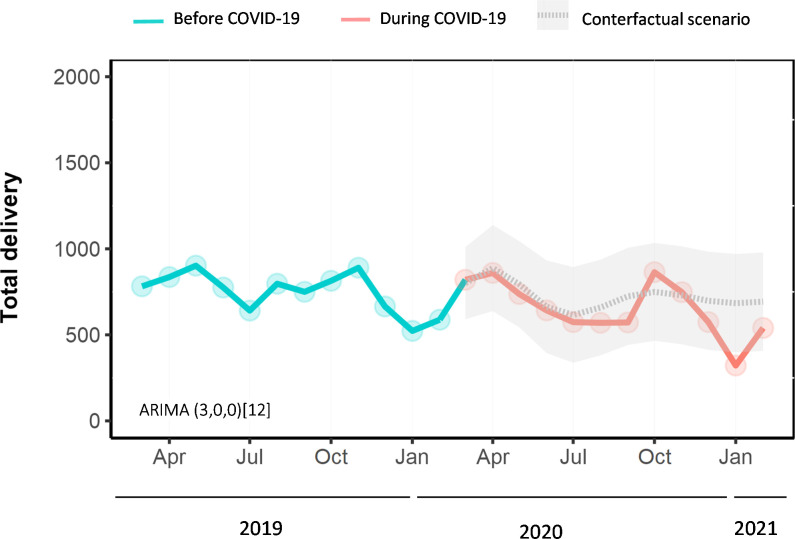
Interrupted time-series analyses of the impact of the COVID-19 pandemic on the total number of deliveries (March 2019 –February 2021) at Tamale Teaching Hospital in Northern Ghana. Note: Observed total delivery (solid lines) was plotted against the counterfactual monthly number of total deliveries predicted by the ARIMA model (grey dashed line) if the COVID-19 pandemic had not occurred. The grey shading depicts 95% prediction intervals. The numbers next to ARIMA in parentheses indicate which components have been included to generate the counterfactual (p,d,q). The square brackets indicate that the model is generated using monthly data (12 months in a year). ARIMA = autoregressive integrated moving average.

Based on our interrupted time series model, on average, there were 1,447 (95% CI -1,841 to -1,054, p<0.001) fewer antenatal care visits per month during the COVID-19 pandemic compared to what would have been expected based on pre-pandemic trends (**Fig 1**). However, after this step change, a slope change suggested an average increase of 81 (95% CI 32.12282 to 129.045, p = 0.001) antenatal care visits per month. The estimated step change for the total number of deliveries suggested an average decrease of 105 deliveries (95% CI -158 to -54, p<0.001) per month during the COVID-19 pandemic compared to what would have been expected based on pre-pandemic trends (**Fig 2**).

**Fig 3:**
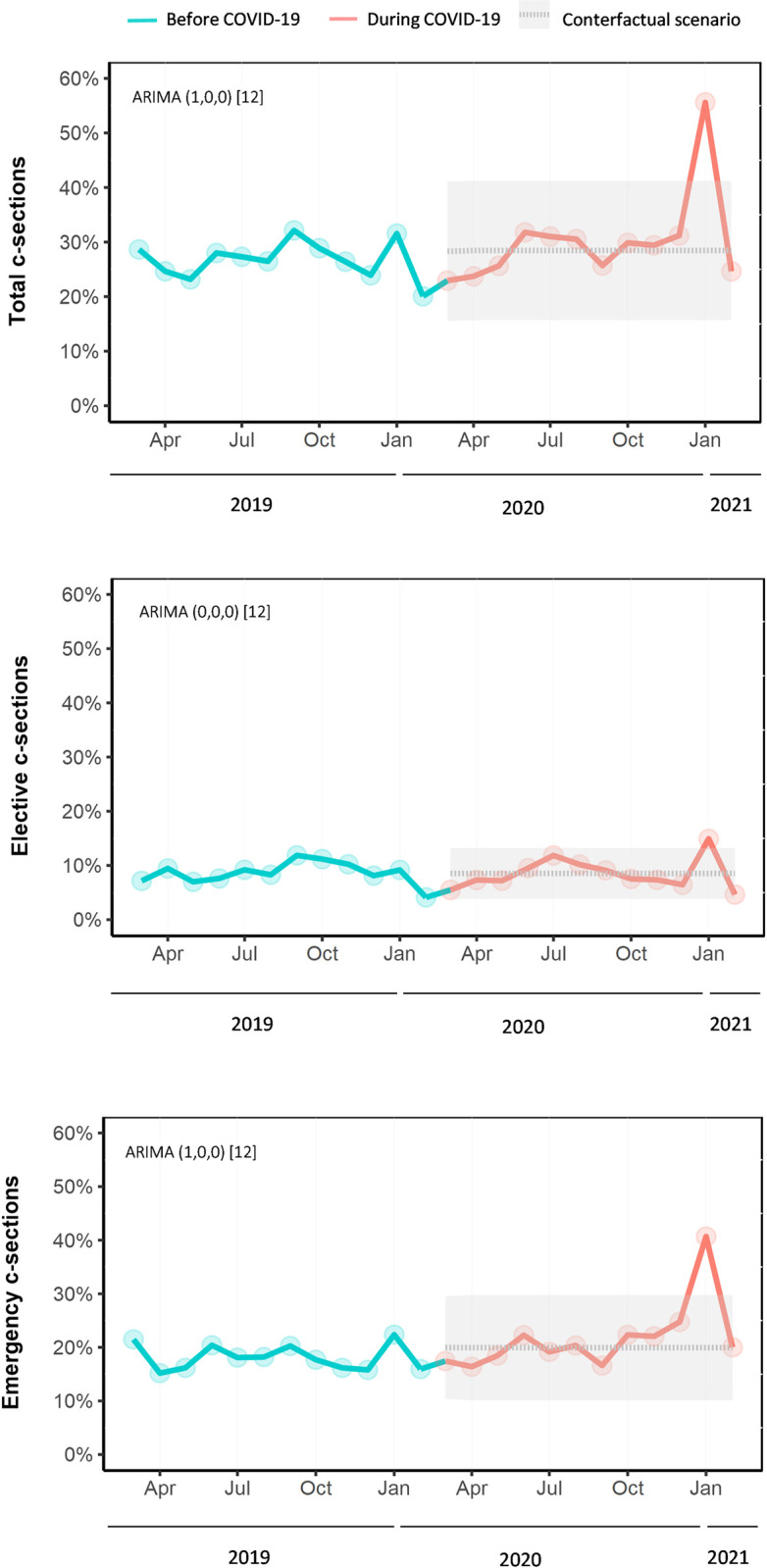
Interrupted time-series analyses of the impact of the COVID-19 pandemic on cesarean sections (March 2019 –February 2021) at Tamale Teaching Hospital in Northern Ghana. Note: Observed cesarean sections (solid lines) were plotted against the counterfactual monthly percentage of cesarean sections from total deliveries predicted by the ARIMA models (grey dashed line) if the COVID-19 pandemic had not occurred. The grey shading depicts 95% prediction intervals. The numbers next to ARIMA in parentheses indicate which components have been included to generate the counterfactual (p,d,q). The square brackets indicate that the model is generated using monthly data (12 months in a year). ARIMA = autoregressive integrated moving average.

On average, there was a 5.4 percent unit decrease (95% CI -10.3% to -0.6%, p = 0.027) in total cesarean sections per month during the COVID-19 pandemic compared to what would have been expected based on pre-pandemic trends (**Fig 3**). However, after this step change, a slope change suggested an average increase of 1.4 percent units (95% CI 0.8% to 2.0%, p<0.001) of total cesarian sections per month. Likewise, on average, there was a 4.0 percent unit decrease (95% CI -10.3% to -0.6%, p = 0.027) in emergency cesarean sections per month during the COVID-19 pandemic compared to what would have been expected based on pre-pandemic trends. However, after this step change, a slope change suggested an average increase of 1.2 percent units (95% CI 0.7% to 1.6%, p<0.001) of emergency cesarean sections per month. Interrupted time series analysis for elective cesarean sections was not statistically significant.

**Fig 4:**
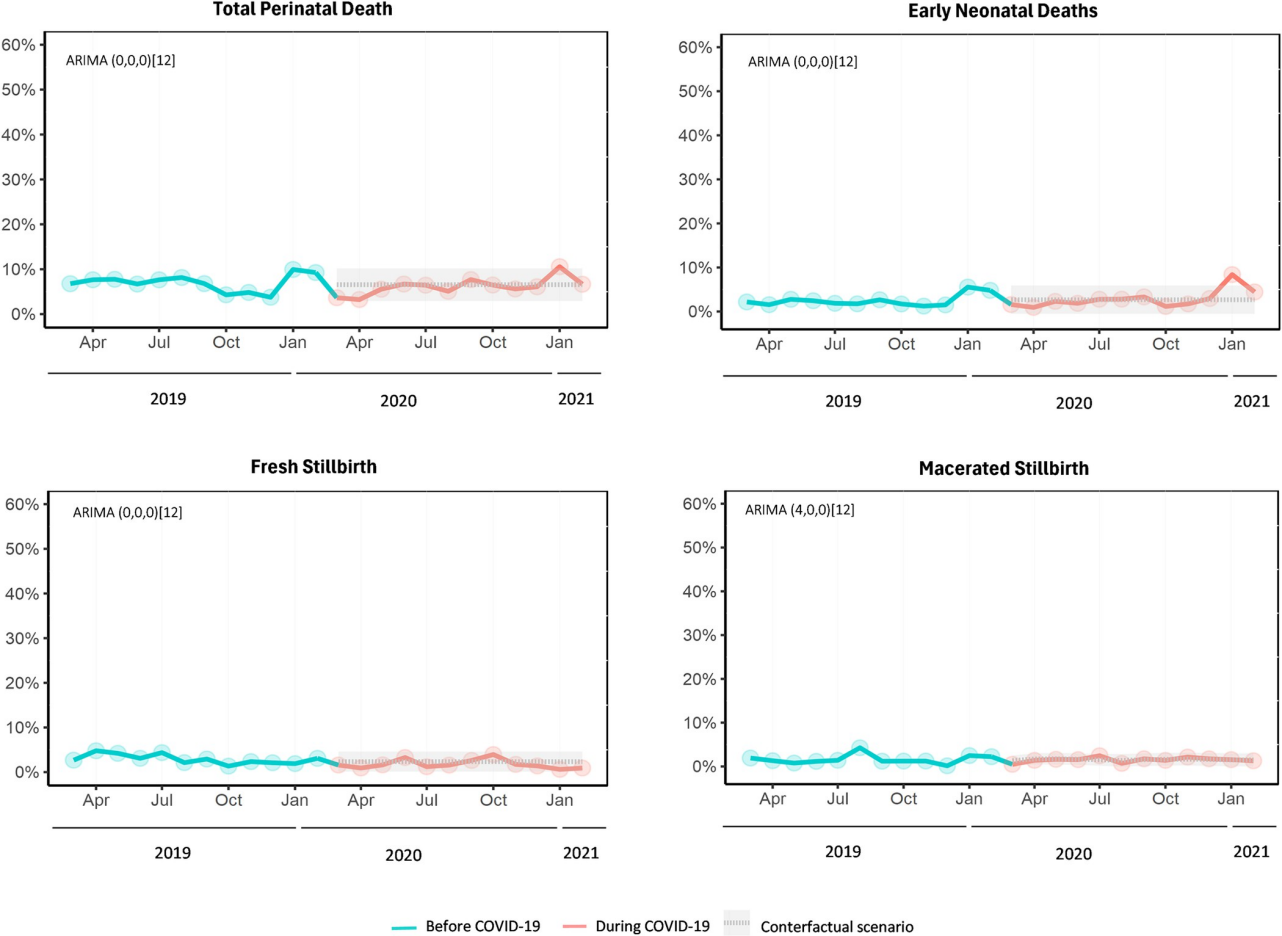
Interrupted time-series analyses of the impact of the COVID-19 pandemic on perinatal death (March 2019 –February 2021) at Tamale Teaching Hospital in Northern Ghana. Note: Observed perinatal deaths (solid lines) were plotted against the counterfactual monthly percentage of perinatal deaths from total deliveries predicted by the ARIMA models (grey dashed line) if the COVID-19 pandemic had not occurred. The grey shading depicts 95% prediction intervals. The numbers next to ARIMA in parentheses indicate which components have been included to generate the counterfactual (p,d,q). The square brackets indicate that the model is generated using monthly data (12 months in a year). ARIMA = autoregressive integrated moving average.

The estimated step change for total perinatal deaths suggested a 3.1 percent unit decrease (95% CI -5.2% to -1.0%, p = 0.005) during the COVID-19 pandemic compared to what would have been expected based on pre-pandemic trends (**Fig 4**). However, after this step change, a slope change suggested an average increase of 0.3 percent units (95% CI 0.08% to 0.6%, p = 0.011) of total perinatal deaths per month. The estimated step change for early neonatal deaths suggested a 1.9 percent unit decrease (95% CI -3.7% to -0.01%, p = 0.049) during the COVID-19 pandemic compared to what would have been expected based on pre-pandemic trends. However, after this step change, a slope change suggested an average increase of 0.3 percent units (95% CI 0.1% to 0.6%, p = 0.003) of early neonatal deaths per month. On average, the estimated step change for fresh stillbirths suggested a 1.1 percent unit decrease (95% CI -1.9% to -0.3%, p = 0.005) during the COVID-19 pandemic compared to what would have been expected based on pre-pandemic trends, while macerated stillbirths showed a pulse change, with an estimated decrease of 1.0 percent units (95% CI -1.9% to -0.1%, p = 0.036) during the COVID-19 pandemic compared to what would have been expected based on pre-pandemic trends.

## Discussion

This hospital-based retrospective study reports the impact of the COVID-19 pandemic on perinatal care and outcomes at the TTH. We found a rapid decline in antenatal care attendance, number of hospital deliveries, cesarean sections, and overall perinatal mortality, with a progressive recovery in most of the cases.

We found the number of deliveries decrease during the COVID-19 pandemic, compared to pre-pandemic trends. Similarly, previous studies documented a decrease in institutional deliveries as an indirect effect of the COVID-19 pandemic [17,23]. For instance, Ashish et al reported a decline in institutional deliveries by almost 50% in Nepal [17]. Fear of infection, lockdown measures and closure of some health facilities have been cited as some of the reasons accounting for the decline in institutional deliveries in Guinea and Liberia during the Ebola pandemic, and Nepal during the COVID-19 pandemic [11,17,24]. Another study among pregnant women during the COVID-19 pandemic reported that women would prefer delivering outside health facilities in order to decrease their risk of infection with the SARS COV-2 virus [25].

The Northern Region of Ghana, where our hospital is located, did not experience a lockdown and delivery services continued without closure. Still, the fear of infection might have been one of the reasons accounting for this decline. The provision of curative services for newborns as well as childhood vaccination services have all experienced declines in our facility [6,26]. The restructuring of antenatal care services, as part of our facility’s pandemic mitigation measures might have also influenced in the reduction of deliveries in our hospital.

Furthermore, we found that the decrease in emergency cesarean sections were an important factor in the decrease of overall cesarean sections during the COVID-19 pandemic. Antenatal care visits play a major role for the assessment of the need of cesarean section in our facility. With reported anxiety among pregnant women and some skipping antenatal care visits because of risk of infection [25], it was not surprising that we recorded fewer antenatal care attendance and subsequently fewer cesarean sections when we compare the pandemic period to the pre-pandemic period. We recorded the lowest level of antenatal attendance in the month of April, 2020. This is possibly due to the increased fear among the population as the country recorded its first cases of COVID-19. The reduction in antenatal care attendance during the pandemic has been well documented [23,27,28]. Kassie et al reported a 27% reduction in antenatal care attendance in Ethiopia [23]. Likewise, in Turkey, a survey by Til et al reported that 4.3% of pregnant women in their study did not receive antenatal care [29].

The TTH instituted a set of measures to curtail the spread of the infection [30]. These measures included strict compliance with social distancing protocols and limiting the antenatal care visits to high-risk pregnancies. These limitations might have also accounted for the decline in antenatal care seen in our study. However, it is worth noting that the number of antenatal care visits increased steadily month by month until almost reaching the numbers seen during the pre-pandemic era in November 2020. This increase was possible due to the revisions made to the hospital’s pandemic response, as more information about the dynamics of the virus became available. One key revision was the assignment of a dedicated team of healthcare workers for pregnancy related COVID-19 cases. This team attended only COVID-19 cases in the Obstetrics department. This measure was stablished with the purpose of reducing the risk of cross infections among staff and increase workforce available to attend antenatal care. Consequently, this approach allowed the hospital to relax restrictions on antenatal visits.

Furthermore, our hospital increased the supply of personal protective equipment such as face masks and coveralls to the frontline workers as the first wave of the pandemic evolved. In light of our experience during the pandemic, we find it critical that facilities evaluate the best measures to allow the continuity of pregnancy monitoring through antenatal care. We also recommend the allocation of specific teams, resources and personal protective equipment for the management of COVID-19-related pregnancies.

This is a foundational study on the impact of the COVID-19 pandemic on perinatal care and outcomes in northern Ghana. However, some limitations warrant discussion. First, this is a hospital-based study and might be prone to selection bias as we only evaluated care and outcomes of pregnant women who reported at our hospital. However, it is important to note that as a tertiary facility, our hospital receives referrals from all peripheral health facilities in the region, and this trend increased during the peak of the pandemic as many peripheral facilities limited the services they provided. Second, our limited monthly data of one year before and after the pandemic is reflected in some of our results, which despite being statistically significant had long confidence intervals, and thus should be interpreted with caution.

## Conclusion

Our findings provide important baseline information regarding the impact of the pandemic on perinatal care and outcomes in northern Ghana. The COVID-19 pandemic had a deleterious effect on perinatal care and outcomes. Pregnancy monitoring through antenatal care should be encouraged and continued even as countries tackle the pandemic.

## Supporting information

S1 Fig Diagnostic plots of interrupted series analysis with ARIMA. A) antenatal visits B) total deliveries C) total cesarean sections D) elective cesarean sections E) emergency cesarean sections F) total perinatal deaths G) early neonatal deaths H) fresh stillbirths I) macerated stillbirths.
(DOCX)

S1 Table Sensitivity analysis and ARIMA model performance. Selected statistically significant models are highlighted in green, whereas selected non-statistically significant models are highlighted in grey.
(DOCX)

S1 Data (XLSX)

S1 File (DOCX)

S2 File (DOCX)
