# Supplementary material for: Impact of the COVID-19 pandemic on perinatal care and outcomes: A retrospective study in a tertiary hospital in Northern Ghana
Source: PLoS One. 2024 May 31;19(5):e0301081. doi: 10.1371/journal.pone.0301081 (PMC11142585; doi:10.1371/journal.pone.0301081)
Supplement: S1 Fig — A) antenatal visits B) total deliveries C) total cesarean sections D) elective cesarean sections E) emergency cesarean sections F) total perinatal deaths G) early neonatal deaths H) fresh stillbirths I) macerated stillbirths. (DOCX) [file pone.0301081.s001.docx]

**S1 Fig. Diagnostic plots of interrupted series analysis with ARIMA.** A) antenatal visits B) total deliveries C) total cesarean sections D) elective cesarean sections E) emergency cesarean sections F) total perinatal deaths G) early neonatal deaths H) fresh stillbirths I) macerated stillbirths

| 1. **Antenatal visits** | 1. **Total deliveries** |
| --- | --- |
| 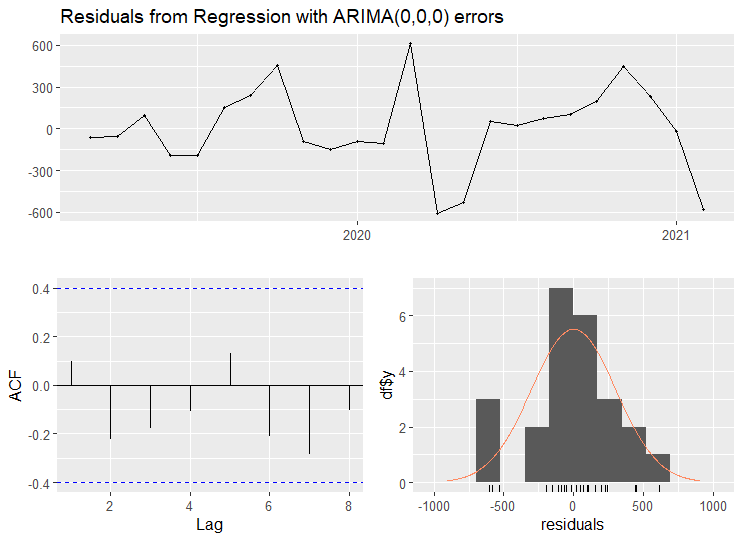 | 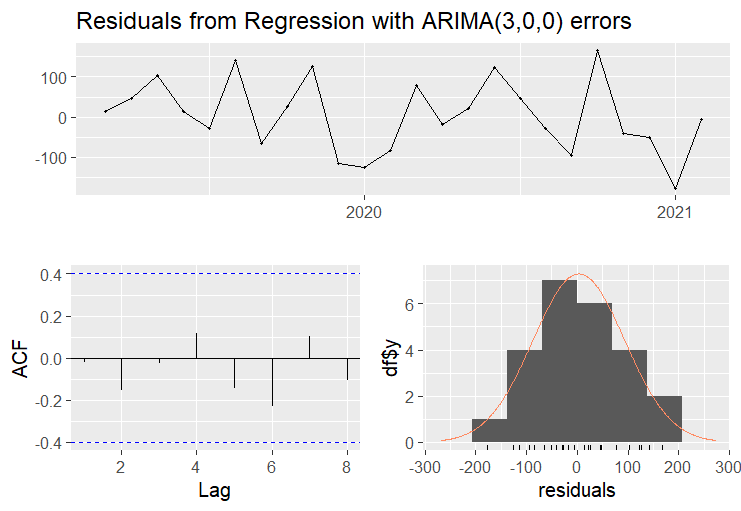 |
| 1. **Total cesarean sections** | 1. **Elective cesarean sections** |
| 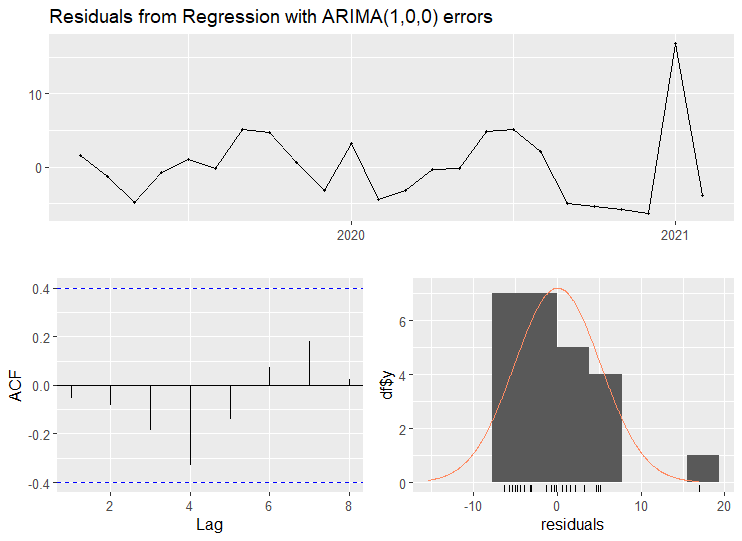 | 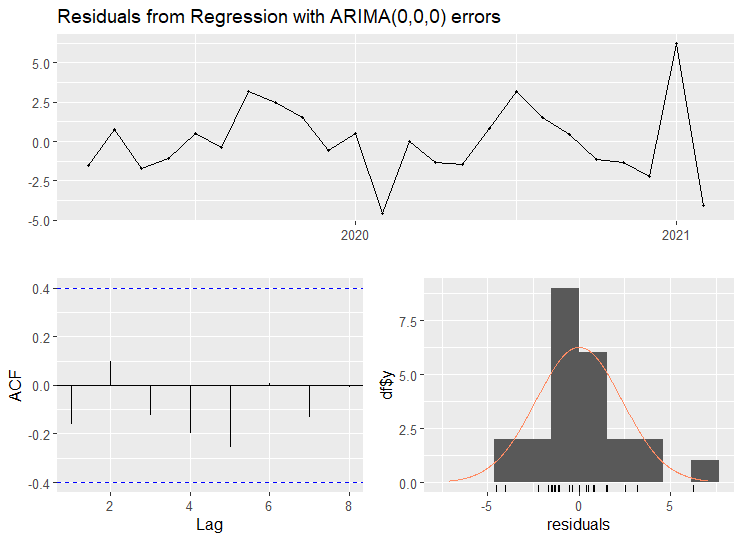 |
| 1. **Emergency cesarean sections** | 1. **Total Perinatal Deaths** |
| 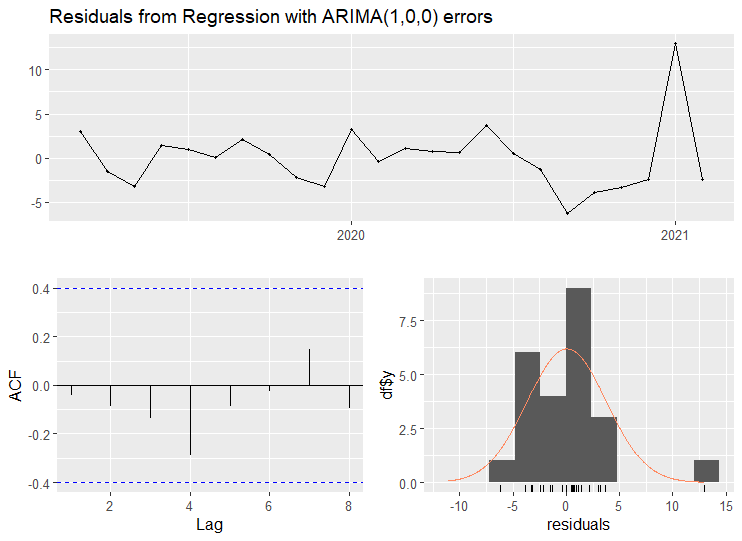 | 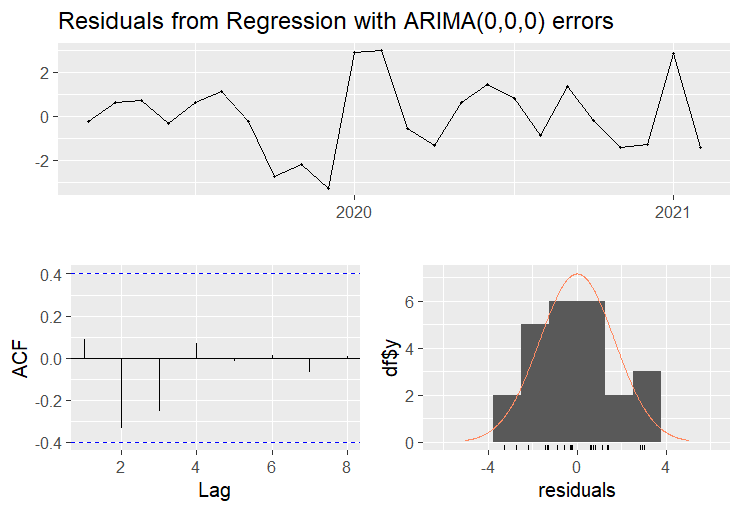 |
| 1. **Early Neonatal Deaths** | 1. **Fresh Stillbirths** |
| 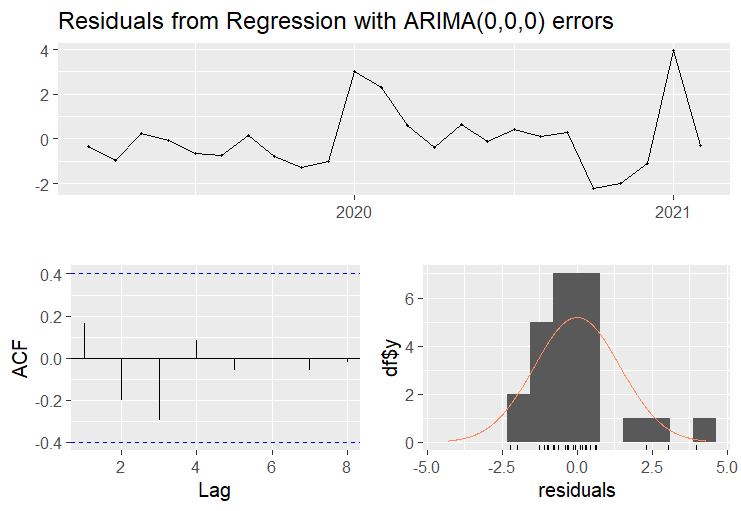 | 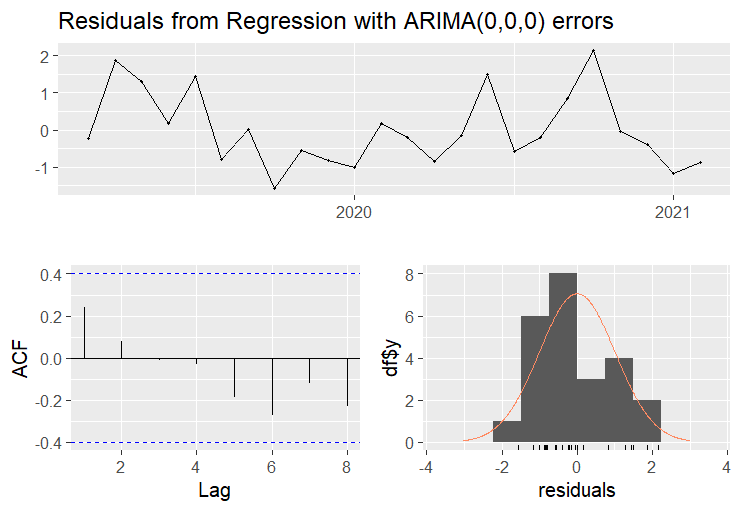 |
| 1. **Macerated Stillbirths** |  |
| 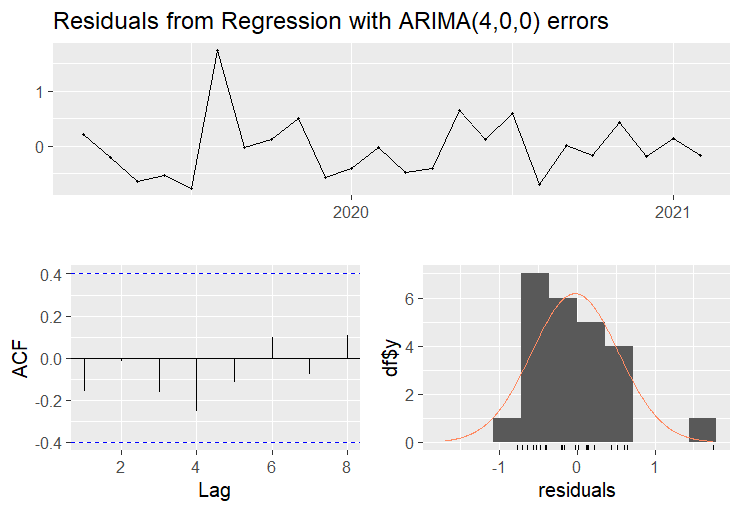 |  |
