# Supplementary material for: Impact of the COVID-19 pandemic on perinatal care and outcomes: A retrospective study in a tertiary hospital in Northern Ghana
Source: PLoS One. 2024 May 31;19(5):e0301081. doi: 10.1371/journal.pone.0301081 (PMC11142585; doi:10.1371/journal.pone.0301081)
Supplement: S1 Table — Selected statistically significant models are highlighted in green, whereas selected non-statistically significant models are highlighted in grey. (DOCX) [file pone.0301081.s002.docx]

**S1 Table.** **Sensitivity analysis and ARIMA model performance.** Selected statistically significant models are highlighted in green, whereas selected non-statistically significant models are highlighted in grey.

| **Counts** | **Step** | | | | **Pulse** | | | | **Step/Ramp** | | | | |
| --- | --- | --- | --- | --- | --- | --- | --- | --- | --- | --- | --- | --- | --- |
|  | AIC | BIC | p-value | CIs including 0 | AIC | BIC | p-value | CIs including 0 | AIC | BIC | p-value step | p-value ramp | CIs including 0 |
| **Antenatal** | 349.4 | 354.1 | 0.000 | no | 339.4 | 342.8 | 0.239 | yes | 349.2 | 353.9 | 0.000 | 0.001 | no |
| **Total deliveries** | 297.3 | 304.4 | 0.000 | no | 291.5 | 297.2 | 0.556 | yes | 295.3 | 303.5 | 0.494 | 0.036 | yes |
| **Perinatal death** | 183.2 | 187.9 | 0.011 | no | 181.6 | 186.1 | 0.442 | yes | 185.0 | 190.9 | 0.046 | 0.639 | yes |
| Fresh stillbirth | 177.5 | 181.0 | 0.006 | yes | 172.5 | 175.9 | 0.937 | yes | 179.1 | 183.8 | 0.232 | 0.548 | yes |
| Macerated stillbirth | 159.5 | 163.0 | 0.370 | yes | 159.0 | 162.5 | 0.239 | yes | 161.5 | 166.2 | 0.646 | 0.921 | yes |
| Early neonatal death | 155.2 | 161.1 | 0.532 | yes | 155.6 | 161.5 | 0.900 | yes | 156.1 | 160.8 | 0.025 | 0.026 | no |
| **Cesarean sections** | 242.9 | 246.5 | 0.393 | yes | 243.6 | 247.2 | 0.867 | yes | 244.4 | 249.1 | 0.928 | 0.446 | yes |
| Elective | 203.6 | 208.3 | 0.693 | yes | 203.7 | 208.5 | 0.985 | yes | 202.6 | 208.5 | 0.412 | 0.088 | yes |
| Emergency | 226.1 | 229.6 | 0.945 | yes | 226.0 | 229.5 | 0.727 | yes | 228.1 | 232.8 | 0.861 | 0.867 | yes |
|  |  |  |  |  |  |  |  |  |  |  |  |  |  |
|  |  |  |  |  |  |  |  |  |  |  |  |  |  |
| **Proportions (variable/total deliveries)** | **Step** | | | | **Pulse** | | | | **Step/Ramp** | | | | |
|  | AIC | BIC | p-value | CIs including 0 | AIC | BIC | p-value | CIs including 0 | AIC | BIC | p-value step | p-value ramp | CIs including 0 |
| **Perinatal death** | 101.1 | 106.9 | 0.280 | yes | 100.1 | 106.0 | 0.139 | yes | 99.9 | 104.7 | 0.005 | 0.011 | no |
| Fresh stillbirth | 73.5 | 77.0 | 0.005 | no | 75.0 | 78.4 | 0.606 | yes | 75.3 | 80.0 | 0.178 | 0.666 | yes |
| Macerated stillbirth | 62.9 | 66.5 | 0.739 | yes | 55.1 | 63.3 | 0.036 | no | 64.5 | 69.3 | 0.479 | 0.526 | yes |
| Early neonatal death | 93.7 | 99.6 | 0.412 | yes | 94.3 | 100.2 | 0.874 | yes | 92.3 | 97.0 | 0.049 | 0.003 | no |
| **Cesarean sections** | 162.4 | 165.9 | 0.185 | yes | 163.3 | 166.8 | 0.377 | yes | 156.0 | 161.9 | 0.027 | 0.000 | no |
| Elective | 116.0 | 119.5 | 0.871 | yes | 114.3 | 117.8 | 0.178 | yes | 117.8 | 122.5 | 0.616 | 0.613 | yes |
| Emergency | 148.4 | 151.9 | 0.064 | yes | 145.9 | 149.3 | 0.783 | yes | 140.3 | 146.2 | 0.031 | 0.000 | no |
