# Supplementary material for: Impact of the COVID-19 pandemic on perinatal care and outcomes: A retrospective study in a tertiary hospital in Northern Ghana
Source: PLoS One. 2024 May 31;19(5):e0301081. doi: 10.1371/journal.pone.0301081 (PMC11142585; doi:10.1371/journal.pone.0301081)
Supplement: S2 File — (DOCX) [file pone.0301081.s005.docx]

**S1 Fig. Diagnostic plots of interrupted series analysis with ARIMA.** A) antenatal visits B) total deliveries C) total cesarean sections D) elective cesarean sections E) emergency cesarean sections F) total perinatal deaths G) early neonatal deaths H) fresh stillbirths I) macerated stillbirths

| 1. **Antenatal visits** | 1. **Total deliveries** |
| --- | --- |
| 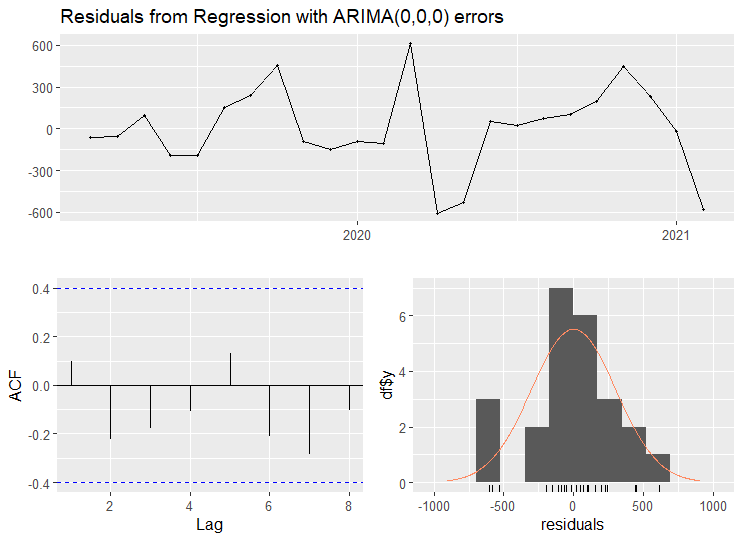 | 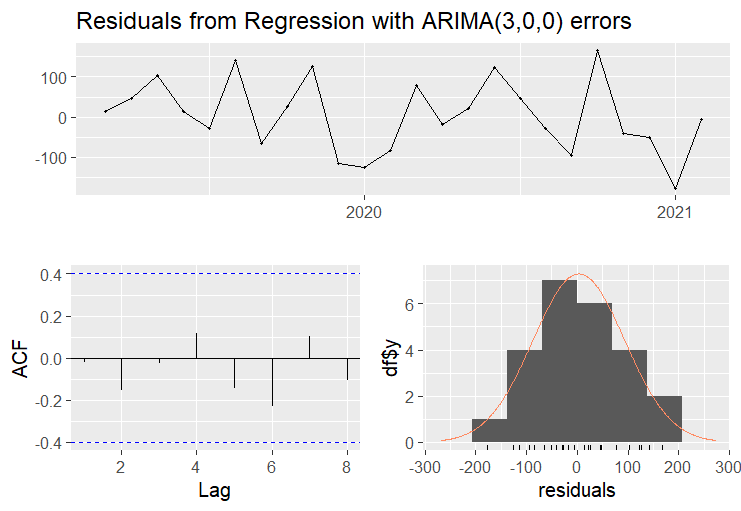 |
| 1. **Total cesarean sections** | 1. **Elective cesarean sections** |
| 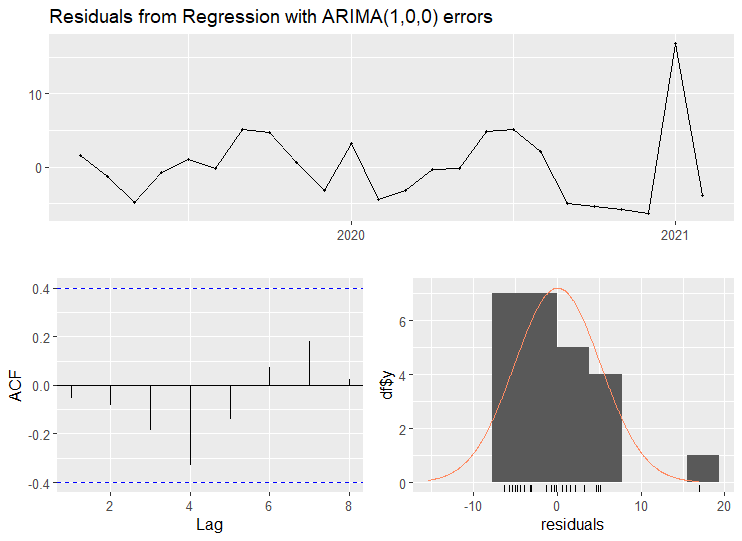 | 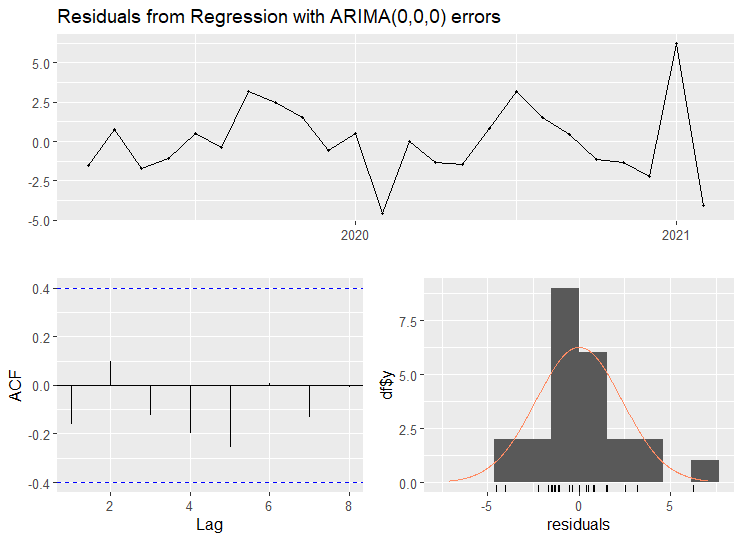 |
| 1. **Emergency cesarean sections** | 1. **Total Perinatal Deaths** |
| 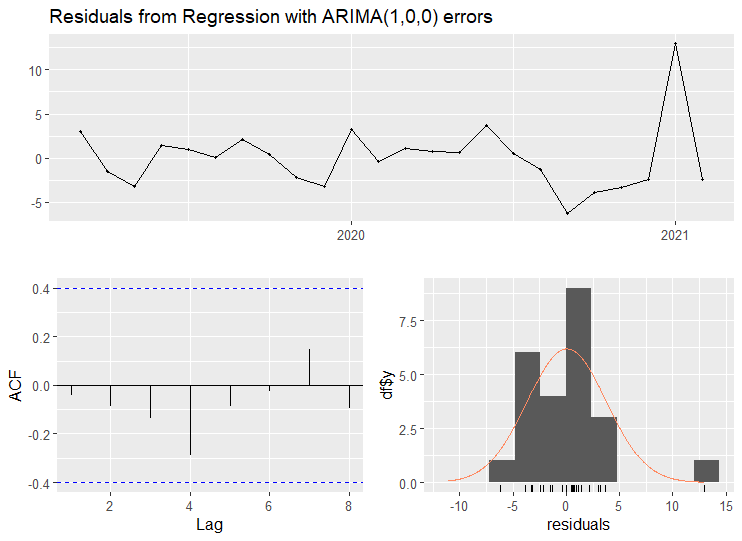 | 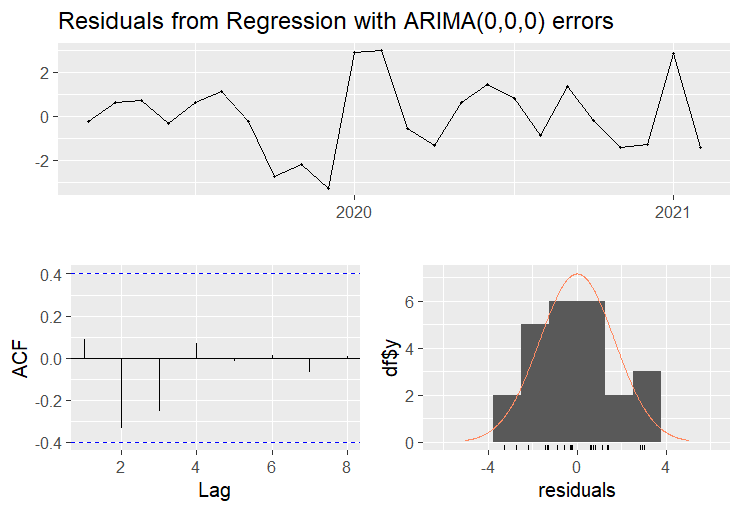 |
| 1. **Early Neonatal Deaths** | 1. **Fresh Stillbirths** |
| 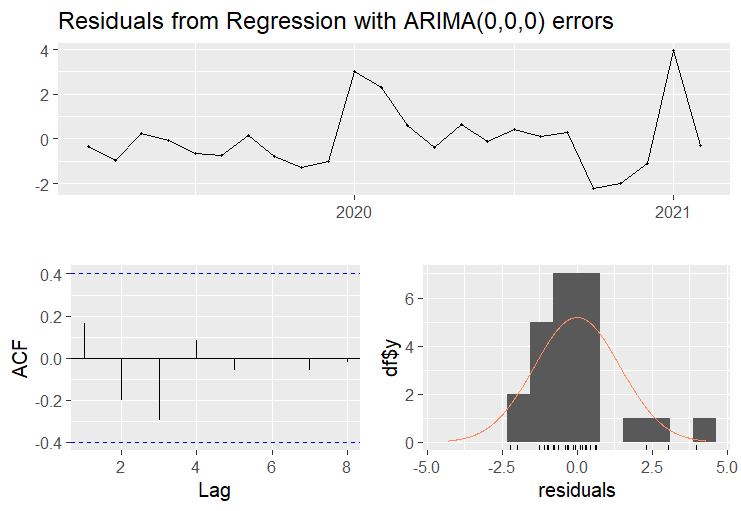 | 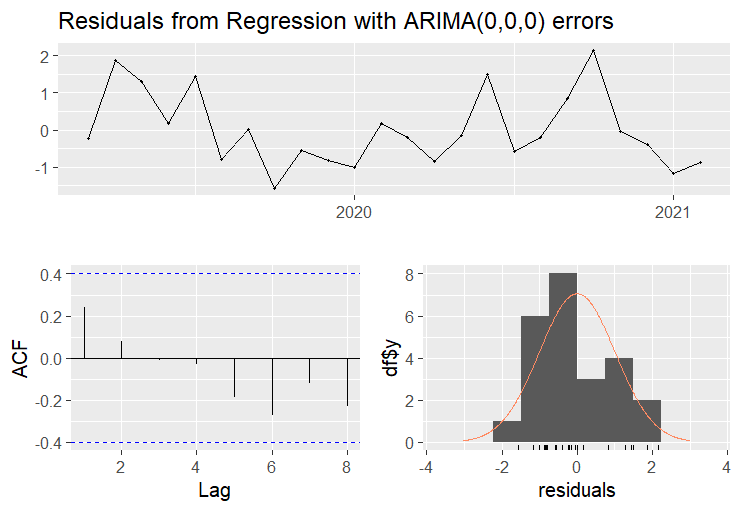 |
| 1. **Macerated Stillbirths** |  |
| 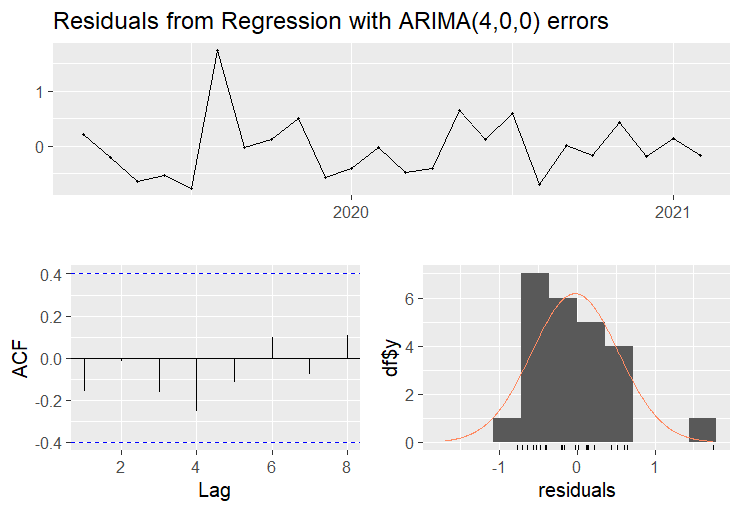 |  |

| **Counts** | **Step** | | | | **Pulse** | | | | **Step/Ramp** | | | | |
| --- | --- | --- | --- | --- | --- | --- | --- | --- | --- | --- | --- | --- | --- |
|  | AIC | BIC | p-value | CIs including 0 | AIC | BIC | p-value | CIs including 0 | AIC | BIC | p-value step | p-value ramp | CIs including 0 |
| **Antenatal** | 349.4 | 354.1 | 0.000 | no | 339.4 | 342.8 | 0.239 | yes | 349.2 | 353.9 | 0.000 | 0.001 | no |
| **Total deliveries** | 297.3 | 304.4 | 0.000 | no | 291.5 | 297.2 | 0.556 | yes | 295.3 | 303.5 | 0.494 | 0.036 | yes |
| **Perinatal death** | 183.2 | 187.9 | 0.011 | no | 181.6 | 186.1 | 0.442 | yes | 185.0 | 190.9 | 0.046 | 0.639 | yes |
| Fresh stillbirth | 177.5 | 181.0 | 0.006 | yes | 172.5 | 175.9 | 0.937 | yes | 179.1 | 183.8 | 0.232 | 0.548 | yes |
| Macerated stillbirth | 159.5 | 163.0 | 0.370 | yes | 159.0 | 162.5 | 0.239 | yes | 161.5 | 166.2 | 0.646 | 0.921 | yes |
| Early neonatal death | 155.2 | 161.1 | 0.532 | yes | 155.6 | 161.5 | 0.900 | yes | 156.1 | 160.8 | 0.025 | 0.026 | no |
| **Cesarean sections** | 242.9 | 246.5 | 0.393 | yes | 243.6 | 247.2 | 0.867 | yes | 244.4 | 249.1 | 0.928 | 0.446 | yes |
| Elective | 203.6 | 208.3 | 0.693 | yes | 203.7 | 208.5 | 0.985 | yes | 202.6 | 208.5 | 0.412 | 0.088 | yes |
| Emergency | 226.1 | 229.6 | 0.945 | yes | 226.0 | 229.5 | 0.727 | yes | 228.1 | 232.8 | 0.861 | 0.867 | yes |
|  |  |  |  |  |  |  |  |  |  |  |  |  |  |
|  |  |  |  |  |  |  |  |  |  |  |  |  |  |
| **Proportions (variable/total deliveries)** | **Step** | | | | **Pulse** | | | | **Step/Ramp** | | | | |
|  | AIC | BIC | p-value | CIs including 0 | AIC | BIC | p-value | CIs including 0 | AIC | BIC | p-value step | p-value ramp | CIs including 0 |
| **Perinatal death** | 101.1 | 106.9 | 0.280 | yes | 100.1 | 106.0 | 0.139 | yes | 99.9 | 104.7 | 0.005 | 0.011 | no |
| Fresh stillbirth | 73.5 | 77.0 | 0.005 | no | 75.0 | 78.4 | 0.606 | yes | 75.3 | 80.0 | 0.178 | 0.666 | yes |
| Macerated stillbirth | 62.9 | 66.5 | 0.739 | yes | 55.1 | 63.3 | 0.036 | no | 64.5 | 69.3 | 0.479 | 0.526 | yes |
| Early neonatal death | 93.7 | 99.6 | 0.412 | yes | 94.3 | 100.2 | 0.874 | yes | 92.3 | 97.0 | 0.049 | 0.003 | no |
| **Cesarean sections** | 162.4 | 165.9 | 0.185 | yes | 163.3 | 166.8 | 0.377 | yes | 156.0 | 161.9 | 0.027 | 0.000 | no |
| Elective | 116.0 | 119.5 | 0.871 | yes | 114.3 | 117.8 | 0.178 | yes | 117.8 | 122.5 | 0.616 | 0.613 | yes |
| Emergency | 148.4 | 151.9 | 0.064 | yes | 145.9 | 149.3 | 0.783 | yes | 140.3 | 146.2 | 0.031 | 0.000 | no |

**S1 Table.** **Sensitivity analysis and ARIMA model performance.** Selected statistically significant models are highlighted in green, whereas selected non-statistically significant models are highlighted in grey.
